# Long-Term Predictors of Social and Leisure Activity 10 Years after Stroke

**DOI:** 10.1371/journal.pone.0149395

**Published:** 2016-02-22

**Authors:** Anna Norlander, Emma Carlstedt, Ann-Cathrin Jönsson, Eva M. Lexell, Agneta Ståhl, Arne Lindgren, Susanne Iwarsson

**Affiliations:** 1 Department of Health Sciences, Lund University, Lund, Sweden; 2 Department of Neurology and Rehabilitation Medicine, Skåne University Hospital, Lund-Malmö, Sweden; 3 Department of Technology and Society, Lund University, Lund, Sweden; 4 Department of Clinical Sciences, Neurology, Lund University, Lund, Sweden; Yokohama City University, JAPAN

## Abstract

**Background:**

Restrictions in social and leisure activity can have negative consequences for the health and well-being of stroke survivors. To support the growing number of people who are ageing with stroke, knowledge is needed about factors that influence such activity in a long-term perspective.

**Aim:**

To identify long-term predictors of the frequency of social and leisure activities 10 years after stroke.

**Method:**

145 stroke survivors in Sweden were followed-up at16 months and 10 years after a first-ever stroke. Data representing body functions, activities & participation, environmental factors and personal factors at 16 months after stroke, were used in multiple linear regression analyses to identify predictors of the activity frequency after 10 years, as assessed by the ‘Community, social and civic life’ sub-domain of the Frenchay Activities Index (FAI-CSC).

**Results:**

At the 10-year follow-up the frequency of social and leisure activities varied considerably among the participants, with FAI-CSC scores spanning the entire score range 0–9 (mean/median 4.9/5.0). Several factors at 16 months post stroke were independently related to the long-term activity frequency. The final regression model included four significant explanatory variables. Driving a car (B = 0.999), ability to walk a few hundred meters (B = 1.698) and extent of social network (B = 1.235) had a positive effect on activity frequency, whereas an age ≥ 75 years had a negative effect (B = -1.657). This model explained 36.9% of the variance in the FAI-CSC (p<0.001).

**Conclusion:**

Stroke survivors who drive a car, have the ability to walk a few hundred meters and have a wide social network at 16 months after a first-ever stroke are more likely to have a high frequency of social and leisure activities after 10 years, indicating that supporting outdoor mobility and social anchorage of stroke survivors during rehabilitation is important to counteract long-term inactivity.

## Introduction

Stroke is one of the primary causes of complex disability in Sweden and globally [1] and is related to extensive long-term costs for healthcare, rehabilitation and productivity loss [2]. In Europe and the U.S. stroke survivors represent 1.5–2.8% of the population [3, 4] and the prevalence is expected to increase [5]. Even though survival rates have improved substantially many stroke survivors experience long-term physical and cognitive disabilities, which often lead to restrictions in activity and social participation [6, 7]. As an effect of the ageing population, improved survival after stroke and a higher incidence of stroke among young people, more people are living and ageing with the consequences of a stroke for a significant part of their lives [8].

In Sweden as well as internationally, specialized stroke care and rehabilitation is mainly concentrated to the first months following the stroke. Most research is also limited to the early post-stroke stages up to the first years of recovery and little is known about the long-term life situation of stroke survivors. As of yet, the few existing follow-up studies that extend beyond five years have mainly focused on survival and disability rates [9, 10]. However, there is an increasing awareness about the need for long-term sustainable stroke services, and long-term studies focusing on participation have been listed among the top ten priorities for stroke research [11]. Two recent 10-year follow-up studies have presented results from stroke survivors, highlighting the need for strategies to reduce the risk of long-term activity limitations [12] as well as the importance of having meaningful activities (such as hobbies and social activities) for subjective well-being 10 years after stroke [13]. Taking into consideration that engagement in social and leisure activities is related not only to subjective well-being but also to improved health, functional recovery and survival after stroke [14–16], research on how to promote such activities in a long-term perspective has not gained sufficient attention.

The initial challenges faced by stroke survivors regarding community reintegration and the process of coping with role changes in family, work and social contexts have been well described [17, 18]. A range of factors have been found related to engagement in social and leisure activities during the first months and years after a stroke. Factors negatively related to engagement in such activities include depression [19], age [20–22], motor and cognitive impairments [21], emotion regulation difficulties [23], living with a partner [20], communication difficulties [6, 20] and urinary incontinence [24]. Whereas walking ability [21] and exercise [25] have been related to higher activity levels. Additional factors described by stroke survivors as facilitators for engagement in valued activities include access to health services and rehabilitation [26], having a meaningful social position or occupation [18, 27, 28] and having social supportive networks [18, 28]. Transportation difficulties [29] and driving cessation [30] are commonly described barriers. However, it is not known what impact these factors have in a longer perspective. Such knowledge could be used to identify those at increased risk for long-term activity limitations and participation restrictions at an early stage, and guide individual rehabilitation interventions and community support that are sustainable over time.

Assessment of engagement in social and leisure activities is challenging since it can be defined in different ways. The present study is based on the widespread and internationally accepted definitions put forth in the International Classification of Functioning Disability and Health (ICF) [31]. In the ICF, activity is defined as the execution of a task or action by and individual. Social and leisure activities are part of the overarching concept ‘community, social and civic life’, defined as: *“actions and tasks required to engage in organized social life outside the family*, *in community*, *social and civic areas of life”*. Execution of such activities implies participation in specific contexts. However, the broader subjective experience of participation, in the ICF defined as involvement in a life situation, was not targeted in this study.

Using these definitions, by means of a 10-year follow-up of Swedish stroke survivors we recently demonstrated that though activity levels generally were high among the participants, there was considerable variation in the frequency of social and leisure activities [32]. A first step in understanding what caused this variation and how to support such activity over time is to identify factors that predict the frequency of social and leisure activities in a long-term perspective. Hence, the aim of this study was to identify long-term predictors of the frequency of social and leisure activities 10 years after a first-ever stroke.

## Methods

### Participants

This study was based on a sample of 416 patients with first-ever stroke consecutively enrolled in the Lund Stroke Register during a one year period starting March 1, 2001. The Lund Stroke Register is a population-based stroke register that covers the catchment area of Skåne University Hospital in Lund, Sweden, including eight municipalities with a total of 234,505 inhabitants (as of December 31, 2001). The efficient case ascertainment methods used to detect patients (including prospective screening methods, regular inquiries to primary care, hospital registers, death and autopsy registers) have been described in previous publications [33, 34]. Stroke was defined according to the established WHO criteria [35]. All eligible patients with an established first-ever stroke during the defined period were included in the study.

**Fig 1:**
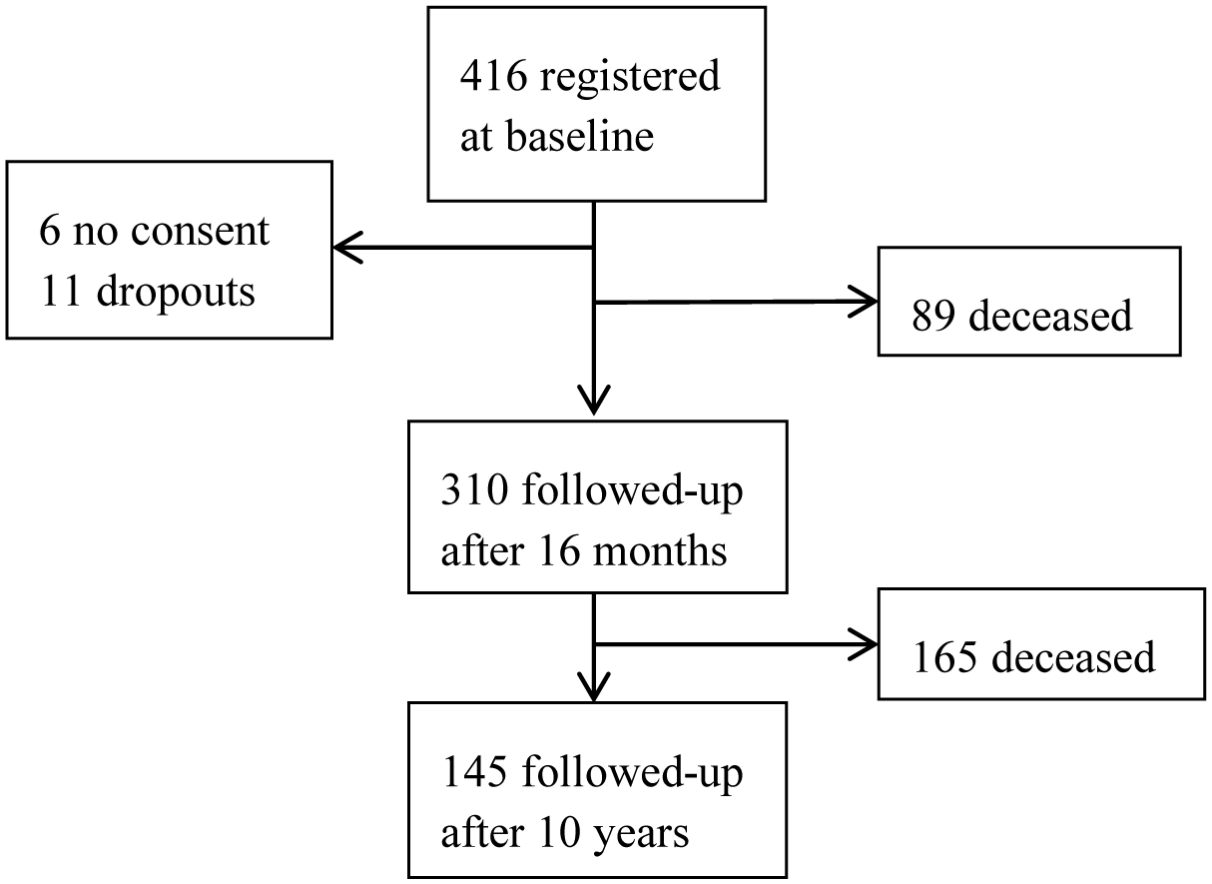
Participant flow chart from inclusion to the 10-year follow-up.

The survivors were followed-up 16 months (SD 0.12) and 10 years (SD 0.17) after the stroke. The 16-month follow-up included 310 participants (89 participants were deceased, 6 declined inclusion at baseline and 11 had dropped out for different personal reasons such as severe illness or the death of a spouse). Beyond this point there were no additional dropouts, and all remaining survivors (n = 145) participated in the 10-year follow-up (Fig 1). Out of the 145 10-year survivors, 59 were women, and the mean age at stroke onset was 66 years. The majority lived in ordinary housing without home care at both follow-ups. Participant characteristics over the study period are presented in Table 1.

**Table 1:** Participant demographics, living situation and functional status at baseline, 16 months and 10 years after stroke (N = 145).

Characteristic	Baseline	16 months	10 years
**Age (years), mean (min-max)**	66 (17–87)	68 (19–88)	76 (28–97)
**Gender (women), n (%)**	59 (41)		
**Education level, n (%)**			
Low (≤ 9 yrs)	86 (59)		
Medium (10–12 yrs)	27 (18)		
High (> 12 yrs/university)	32 (22)		
**Stroke type, n (%)**			
Cerebral infarction	126 (87)		
Intracerebral hemorrhage	10 (7)		
Subarachnoid hemorrhage	8 (5)		
Undefined	1 (1)		
**Stroke severity (NIHSS) median (Q**_**1**_**-Q**_**3**_**)**	3.0 (1.5–5.0)		
**Recurrent stroke since baseline, n (%)**		8 (6)	22 (15)
**Housing situation, n (%)**			
Ordinary housing, no home care		131 (90)	99 (68)
Ordinary housing, with home care		11 (7)	31 (22)
Assisted living facility/residential care		3 (2)	15 (10)
**ADL dependence (Barthel Index)**^1^, **n (%)**			
Independent		124 (86)	106 (73)
Moderate dependence		15 (10)	19 (13)
Major dependence		6 (4)	20 (14)
**Overall activity level (mFAI)**^2^, **n (%)**			
Inactive			34 (23)
Moderately active			42 (29)
Highly active			69 (48)

NHISS: The National Institutes of Health Stroke Scale, score range 0–42 [36].

mFAI: Swedish modified and extended Frenchay Activities Index [37].

^1^ Independence = 95–100, moderate dependence = 60–90, major dependence = 0–55 [38].

^2^ Inactive = 0–16; Moderately active = 17–32; Highly active = 33–48.

### Procedure

At the 16-month as well as the 10-year follow-up the majority of the participants were followed-up at the outpatient clinic of the Department of Neurology and Rehabilitation Medicine, Skåne University Hospital, Lund. The remaining follow-ups were performed at other care facilities, at home visits, or by telephone, and in some cases in cooperation with primary care professionals in the district where the participant lived at the time of the follow-up. All assessments at the 16-month as well as the 10-year follow-up were performed by the same researcher (A J; a specialist nurse). With the exception of the few participants who were followed-up by telephone, all assessments were administered by means of face to face interviews. If participants had difficulties to answer the questions because of cognitive, communicative or health-related problems they were assisted by a relative or caregiver who knew the participant well. For further details on procedures and assessment methods we refer to our previous publications [34, 39].

#### Instruments and study-specific questions

The data used for the present study included a subset of the extensive data collected at the 16-month and the 10-year follow-ups. At the 16-month follow-up, different aspects of stroke outcome were obtained using well-established assessment instruments as well as study-specific questions. Assessment instruments included the Barthel Index (BI) [40], Mini Mental State Examination (MMSE) [41], Geriatric Depression Scale (GDS-20) [42] and the Medical Outcomes Study 36-Item Short-Form Health Survey (SF-36) [43]. The study-specific questions covered lifestyle related risk factors, social network resources [44], satisfaction with stroke services and rehabilitation, stroke related functional disabilities, pain, housing situation, activities of daily living (ADL), indoor and outdoor mobility and general health. A majority of these questions were based on the items of the Swedish Stroke Register 2-year follow-up survey [45].

At the 10-year follow-up, information about activity frequency was obtained by means of a Swedish extended version of the Frenchay Activities Index (FAI) [37]. FAI assessments are based on self-reported frequency of performing different activities over a period of time. The instrument has demonstrated adequate validity and reliability in populations with stroke [46, 47] and is commonly used in stroke research to assess social activity [7, 14, 20]. However, by means of ICF-linking we recently demonstrated [32] that only a sub-domain of the FAI is related to the ICF chapter ‘Community, social and civic life’ whereas the remaining items mainly represent domestic activities and mobility. Hence, for the present study only the ‘Community, social and civic life’ sub-domain of the FAI, hereinafter referred to as FAI-CSC, was used.

#### Dependent variable

The FAI-CSC, representing the frequency of social and leisure activities 10 years after stroke, consisted of three items; “Social outings” (frequency of taking part in social activities out of home, such as going to the theater, dinner with friends or visiting family), “Pursuing active interest in hobby” (activities of interest in or out of the home, such as knitting, caring for houseplants or sports) and “Outings/car rides” (coach or rail trips or car rides to some place for pleasure). Even though an extended version of the FAI was used in the data collection, the three items of the FAI-CSC were identical to those of the original FAI [47]. For the first two items, frequency of performance during the last three months was assessed whereas the last item referred to the previous six months. Each item was scored from 0–3, giving a maximum total score of 9 for the FAI-CSC. A higher FAI-CSC score reflected a higher overall frequency of social and leisure activities.

#### Independent variables

Since engagement in social and leisure activities is a complex process likely influenced by many different factors over time, it was relevant to include a broad range of independent variables representing different aspects of functioning, disability and health, described in the ICF in terms of four components; Body functions, Activities and participation, Personal factors and Environmental factors [31]. A total of 22 independent variables were selected from the comprehensive assessments performed at the 16-month follow-up. The selection was guided by previous research findings reviewed by the first two authors, followed by repeated discussions in the interdisciplinary team of co-authors. The variables were divided into four groups, representing Body functions (8 variables), Activities and participation (6 variables), Personal factors (2 variables) and Environmental factors (6 variables) (Table 2). Before further analysis, the GDS-20 and the MMSE were dichotomized according to established cut-off levels for depressive mood [42] and cognitive impairment [48]. Four other variables (impaired motor function; age; main occupation; particular person for support) that had numerous or overlapping response categories were dichotomized or recoded to improve interpretability. The recoding was based on the type of response categories and the distribution of data.

**Table 2:** Independent variables assessed at 16 months post stroke and their correlation with the frequency of social and leisure activity (FAI-CSC) 10 years after stroke (N = 145).

Independent variables	n (%)	FAI-CSC Md (q_1_-q_3_)	Spearman r	p*
**BODY FUNCTIONS**				
**Indication of cognitive impairment (MMSE)**^1^				0.025
Yes (<25)	11 (7.9)	3 (0–4)		
No (≥25)	129 (92.1)	6 (3–7)		
**Indication of depressive mood (GDS-20)**^2^				0.005
Yes (>5)	43 (30.1)	3 (1–7)		
No (≤5)	100 (69.9)	6 (4–7.8)		
**Impaired motor function rel. to stroke**^3^				0.002
No	81 (56.3)	6 (4–7)		
Yes, upper extremities	21 (14.6)	5 (2.5–8)		
Yes, lower extremities	12 (8.3)	6 (3.5–8)		
Yes, both upper and lower extremities	30 (20.8)	3 (0–5)		
**Do you experience difficulty speaking?**^3^				0.073
No	127 (88.2)	6 (3–7)		
Yes	17 (11.8)	4 (2.5–5.5)		
**Do you easily get angry?**^3^			0.141	0.093
Almost never	87 (60.4)	5 (2–5)		
Sometimes	46 (31.9)	6.5 (3-()		
Often	11 (7.6)	4 (3–7)		
Constantly	0			
**Do you experience any pain?**^3^			-0.131	0.117
Almost never	78 (54.2)	6 (3–7)		
Sometimes	36 (25)	5 (3–8)		
Often	20 (13.9)	4 (0.3–8)		
Constantly	10 (6.9)	2.5 (0–6.5)		
**Do you feel tired?**^3^				
Almost never	26 (18.1)	6 (4.8–7)	-0.120	0.153
Sometimes	76 (52.8)	5 (3–7)		
Often	28 (19.4)	4 (1.3–8)		
Constantly	14 (9.7)	4.5 (2.3–7.3)		
**Urinary incontinence (BI item 6 “Bladder”)**			0.239	0.004
Incontinent	7 (4.8)	1 (1–2)		
Occasional accident	13 (9.0)	5 (1–6)		
Continent	125 (86.2)	6 (3–7.5)		
**ACTIVITIES AND PARTICIPATION**				
**Do you drive a car?**^3^				<0.001
Yes I drive	86 (59.7)	6 (4–8)		
No, but did before stroke	24 (16.7)	3 (1–6)		
No, and not before stroke	34 (23.6)	3.5 (0–5.3)		
**Do you use public transport?**^3^				0.563
Yes	50 (34.7)	6 (3.8–7)		
No	94 (65.3)	5 (3–8)		
**How often do you exercise (walking, skiing, bicycling, swimming, sports, running)?**^3^^,^^a^			0.255	0.002
Never	16 (11.1)	2 (0.3–4.5)		
< 1/week	4 (2.8)	6 (1–8.8)		
1/week	6 (4.2)	3 (0–7)		
2–3/week	31 (21.5)	6 (3–7)		
Almost every day	87 (60.4)	6 (4–8)		
**How mobile are you?**^3^			-0.267	0.001
Independent indoors and outdoors ^c^	132 (91.7)	6 (3–7)		
Independent indoors but not outdoors ^c^	5 (3.5)	3 (0–4.5)		
Dependent both indoors and outdoors	7 (4.9)	1 (0–3)		
**Are you able to carry out your pre-stroke interests?**^3^			-0.327	<0.001
Yes, as before	74 (51.4)	6 (4–8)		
Yes, but not quite as before	49 (34)	5 (2–7,5)		
No, hardly or never	21 (14.6)	3 (0.5–4.5)		
**Does your health condition limit your ability to walk a few hundred meters? (SF36-3h)**^3^			0.436	<0.001
Yes, very limited	12 (8.3)	1 (0.3–4.5)		
Yes, slightly limited	25 (17.4)	3 (0–5)		
No, not at all limited	107 (74.3)	6 (4–8)		
**PERSONAL FACTORS**				
**Age**				<0.001
< 75 yrs	107 (73.8)	6 (4–8)		
≥ 75 yrs	38 (26.2)	2 (0–4.3)		
**Gender**				0.052
Men	86 (59.3)	6 (3–8)		
Women	59 (40.7)	4 (1–7)		
**ENVIRONMENTAL FACTORS**				
**Living situation**				0.160
Living alone	36 (24.8)	4 (1.3–6.8)		
Living with partner or other person(s)	109 (75.2)	6 (3–7)		
**Main occupation**				0.186
Work/study fulltime or part time	27 (18.6)	6 (4–7)		
Do not work/study	118 (81.4)	5 (2–7)		
**Is there any particular person that you feel you can truly get support from?** ^3^				0.924
Yes	134 (93.1)	5 (3–7)		
No	10 (6.9)	4.5 (3–8.3)		
**Do you receive any rehabilitation/training at present?**^3^				0.236
Yes	27 (18.8)	4 (3–6)		
Yes, but not enough	4 (2.8)	3 (0–6)		
No, but I would need it	20 (13.9)	4 (3–7.5)		
No, I do not need it	93 (64.6)	6 (3–7.5)		
**Social anchorage outside the household (contact frequency)**^3^^,^^b^			0.252	0.002
Daily	47 (32.4)	6 (4–8)		
Every week	92 (63.4)	5 (2.3–7)		
Every month	3 (2.1)	0 (0–0)		
Every quarter of a year	1 (0.7)	4 (4–4)		
Never	1 (0.7)	0 (0–0)		
**Social anchorage outside the household (extent of social network, 0–5 sources)**^3^^,^^b^			0.369	<0.001
5 different sources of contact	48 (33.3)	6.5 (4.3–8)		
4 different sources of contact	44 (30.6)	6 (3–8)		
3 different sources of contact	39 (27.1)	3 (1–6)		
2 different sources of contact	8 (5.6)	4.5 (1–6.8)		
1 different sources of contact	4 (2.8)	1.5 (0–3.8)		
None	1 (0.7)	0 (0–0)		

FAI-CSC: The ‘Community, social and civic life’ sub-domain of the Frenchay Activities Index (score range 0–9) [32]. MMSE: Mini Mental State Examination [41]. GDS-20: Geriatric Depression Scale [42]. SF36: Medical Outcomes Study 36-Item Short-Form Health Survey [43]. BI: Barthel ADL Index [40].

Unless otherwise indicated, the questions are based on the Swedish Stroke Register (Riksstroke) 2-year follow-up survey [45].

^a^ Part of a protocol inspired by Lindström et al 2001 [49].

^b^ Question based on Hanson & Östergren 1987 [44].

^c^ With or without mobility devices.

^1^ 5 missing.

^2^ 2 missing.

^3^ 1 missing (due to language difficulties, severe aphasia, health problems or unwillingness to do the test).

* For dichotomized or nominal variables the p-value refers to the Kruskal Wallis test for difference between groups, and for ordinal variables the p-value refers to the Spearman test for correlation.

#### Statistical analyses

The independent variables listed in Table 2 were first tested individually for their association with the FAI-CSC, using the Spearman rho correlation analysis for ordinal data and the Kruskal-Wallis test for nominal data. The variables that reached the pre-defined statistical significance level of p≤0.25 qualified for further investigation using multiple linear regression analyses. In order to fit reasonable regression models all independent variables were dichotomized. Dichotomizations were determined for each variable based on its response categories and the distribution of data. The dichotomized variables were then categorized in accordance with the four ICF-components (Body functions, Activities and participation, Personal factors and Environmental factors) and one regression model created for each component, all with the FAI-CSC as the dependent variable. The regression models were reduced manually using a stepwise backward method, until only significant (p<0.05) variables remained. Finally, the independent variables that were identified as statistically significant predictors in each of the four separate regression models were included in a combined model, using the same stepwise backward method as before (p<0.05). This final model was also controlled for potential confounders in terms of stroke severity, stroke type, cardiac disease, recurrent stroke and pre-stroke education level. Residuals of the final regression model were tested for normality using the Shapiro-Wilks test. Participants with missing data (see Table 2) were excluded but remained in all analyses where data was available. All analyses were undertaken using the IBM SPSS 22 software.

#### Ethics

The Lund Stroke Register has been approved by the Ethics Committee of Lund University or the Regional Ethical Review Board in Lund several times related to different studies. The 10-year follow-up was approved in May 2011 (No. 2011/278). Written informed consent was obtained from each participant or next of kin.

## Results

The variation in FAI-CSC scores for the total sample spanned the entire score range (0–9), with a mean score of 4.86 (SD 2.83). With the exception of two variables (use of public transport; having a particular person for support), the correlation analyses showed that most of the selected variables from the 16-month follow-up were related to the FAI-CSC score 10 years after stroke (Table 2). The variables that qualified for inclusion in the multiple regression analyses consisted of eight variables related to body functions, five related to activities and participation, two to personal factors, and five to environmental factors (Table 3). Among the eight independent variables of the body functions model, impaired motor function in both upper and lower extremities significantly predicted a lower frequency of social and leisure activities after 10 years. In the activities and participation model, driving a car and having the ability to walk a few hundred meters predicted a more positive 10-year outcome. Age was the single significant predictor in the personal factors model, where an age of 75 years or above was related to a lower frequency of social and leisure activities. In the model for environmental factors, having a wide social network predicted a more favorable 10-year outcome. The significant predictors identified in these four separate regression models are presented in Table 4. The activities and participation model showed the highest explanatory power (R^2^ = 27.3%).

**Table 3:** The dichotomized independent variables included in the multiple regression analyses.

BODY FUNCTIONS	ACTIVITIES AND PARTICIPATION	PERSONAL FACTORS	ENVIRONMENTAL FACTORS
**Indication of cognitive impairment (MMSE)**	**Driving a car**	**Age**	**Living situation**
0) No (≥25)	0) No	0) <75 years	0) Living alone
1) Yes (<25)	1) Yes	1) ≥75 years	1) Living with partner or other person(s)
**Indication of depressive mood (GDS-20)**	**Exercise frequency**	**Gender**	**Main occupation**
0) No (≤5)	0) ≤ 1/week	0) Women	0) Do not work/study
1) Yes (>5)	1) >1/week	1) Men	1) Work/study fulltime or part time
**Impaired motor function**	**Mobility**		**Rehabilitation/training**
0) No impairment, or only upper or lower extremity	0) Independent indoors and outdoors		0) Unmet perceived need
1) Impaired function in both upper and lower extremities	1) Dependent outdoors or both indoors/outdoors		1) Perceived need met
**Speaking difficulties**	**Ability to carry out pre-stroke interests**		**Social contact frequency**
0) No	0) No, hardly or never		0) Less than daily
1) Yes	1) Yes, as before or almost as before		1) Daily
**Anger**	**Ability to walk a few hundred meters**		**Extent of social network**
0) No	0) Very or slightly limited		0) 0–3 sources of contact
1) Yes	1) Not at all limited		1) 4–5 sources of contact
**Anger**			
0) Almost never or sometimes			
1) Often or constantly			
**Pain**			
0) Almost never or sometimes			
1) Often or constantly			
**Tiredness**			
0) Almost never or sometimes			
1) Often or constantly			
**Urinary incontinence**			
0) Continent or occasional accident			
1) Incontinent			

MMSE: Mini Mental State Examination [41]. GDS-20: Geriatric Depression Scale [42].

**Table 4:** ICF-component specific predictors of social and leisure activity frequency 10 years after stroke (N = 145).

ICF components	Predictors*	B	95% CI	p	R^2^ (%)
**Body functions**	Impaired motor function^1^	-2.351	-3.439; -1.263	<0.001	11.4
**Activities and participation**	Driving a car^2^	1.602	0.724; 2.479	<0.001	27.3
	Ability to walk a few hundred meters^3^	2.295	1.310; 3.280	<0.001	
**Personal factors**	Age (≥ 75 yrs)	-2.585	-3.556; -1.615	<0.001	16.2
**Environmental factors**	Extent of social network^4^	2.161	1.251; 3.070	<0.001	13.4

* Independent predictors identified by multiple regression analyses for each ICF-component.

^1^ 0 = No impairments/impaired function in either upper or lower extremity, 1 = Impaired function in both upper and lower extremities.

^2^ 0 = Does not drive (includes both those who drove before stroke and those who did not), 1 = Drives.

^3^ 0 = Slightly or very limited, 1 = Not at all limited.

^4^ 1 = 0–3 different sources, 2 = 4–5 different sources.

When the significant variables from all four models were combined into a final multiple linear regression model, four variables remained significant (Table 5). Driving a car (B = 0.999, p = 0.024), ability to walk a few hundred meters (B = 1.698, p = 0.001) and extent of social network (B = 1.235, p = 0.004) had a positive effect on the outcome, whereas age had a negative effect (B = -1.657, p = <0.001). The explanatory power (R^2^) of the final model was 36.9%. When tested for potential confounders (stroke severity, stroke type, cardiac disease, recurrent stroke, education level) no significant change was detected in the coefficients of the final model. Residuals in the final regression model were normally distributed (Shapiro-Wilks test p = 0.484).

**Table 5:** Independent predictors of social and leisure activity frequency 10 years after stroke (N = 145).

Predictors*	B	95% CI	p
Driving a car	0.999	0.135; 1.863	0.024
Ability to walk a few hundred meters	1.698	0.738; 2.658	0.001
Extent of social network	1.235	0.396; 2.074	0.004
Age (≥75 yrs)	-1.657	-2.576; -0.738	<0.001

*Based on the combined regression model including all four ICF-components.

Explanatory power (R^2^) = 36.9%.

## Discussion

The results of this follow-up study demonstrate that a wide range of factors assessed during the second year after a first-ever stroke is related to the frequency of social and leisure activities 10 years after the stroke. Some key factors, including car-driving, walking ability, social network and age, seem to be especially indicative of the long-term activity frequency.

Two out of the four final predictors identified in this study (i.e. ‘Ability to walk a few hundred meters’ and ‘Driving a car’) represent out-of-home mobility. These results could be explained by the fact that the FAI-CSC mostly consists of out-of-home activities that may require some transportation. Walking distance has previously been described as central for activity engagement both within home and in the community [50]. Similarly, several studies have shown that loss of driver’s license after stroke has profound implications including restricted community participation and social isolation [30, 51]. However, our results also suggest that the initial difficulties related to getting around after the stroke have been insufficiently compensated for over time. To avoid long-term inactivity, community accessibility is an important issue to address, especially considering that 26% of all stroke survivors in Sweden, and 40% of those over the age of 75, report dependence on others in outdoor mobility one year after stroke [52]. White and co-workers [53] highlighted the importance of providing driving assessments and information about the process of returning to driving or alternatives such as public transport prior to hospital discharge and at follow-ups. Following discharge, community-based interventions should adress stroke survivors’ ability to get around outside home using different modes of transportation.

We also found that an age of 75 years or older at the 16 months follow-up predicted a lower frequency of social and leisure activities 10 years after the stroke. This is not surprising considering that engagement in leisure activities generally decreases with age [54]. Ageing with a disability may also lead to accelerated physical and cognitive impairments [55] and restrictions in community participation [56]. In addition, high age is a risk factor for more severe strokes [57]. However, even after controlling our final model for stroke severity the independent effect of age on the long term activity level remained. Accordingly, our results indicate that stroke survivors over the age of 75 are a risk group for social and leisure inactivity that deserves special attention in stroke management and rehabilitation.

Another factor found to influence the 10-year activity frequency was the social network that the participants reported having at the 16-month follow-up. Having supportive social networks has previously been linked to improved physical [58] and psychological [59] recovery after stroke, and has been described as an important factor for community reintegration and participation [18]. Difficulties to maintain and acquire new social relationships after stroke have also been described [18]. Out of the three variables related to the social network included in our analyses (representing the number and frequency of social contacts and whether the participants perceived that they had a particular person for support), the number of contacts was the only significant predictor of the long-term activity frequency. A wide social network may have acted as a buffer against social isolation over time and provided support which was beneficial for the long-term recovery and social reintegration. It may also be a reflection of the social activity level the participants had before their stroke, which is supported by ageing research demonstrating previous activity habits as a strong predictor of activity later in life [54]. Unfortunately, we did not have access to information about the participants’ social and leisure activity before the stroke or at the 16-month follow-up, which may have provided additional insights.

Even though most current stroke rehabilitation interventions are aimed at restoring body functions, our results indicate that such functions are not the most important predictors for the long-term activity frequency. However, it should be kept in mind that the 10-year survivors in this study on average were relatively young and had less severe strokes. Whether body functions would turn out as significant predictors in samples with more severe disabilities remains to be investigated. It could also be that long-term stroke survivors have adapted to and compensated for their impairments over time. The ability of the person to accept and adapt to their stroke-related problems have been found as central factors to engagement in social activities [18] but research exploring the influence of such processes in a long-term perspective is needed.

### Methodological considerations

The population-based sample with low loss to follow-up and no dropouts from the 16-month to the 10-year follow-up is a noteworthy strength of the present study. The strong retention of participants was likely thanks to the great efforts put into localizing and contacting the survivors, as well as the participants’ previous contacts with the researcher who performed all follow-ups. To avoid exclusion of survivors with cognitive or communicative difficulties, proxy respondents were used which may have influenced the results. Previous studies have demonstrated adequate agreement between patients and proxy respondents in assessment of risk factors as well as activity measures after stroke and support the use of proxies to reduce sample selection bias [60, 61]. When needed, the presence of spouses or caregivers who knew the participants well was considered to strengthen the validity of the results by helping the participants to provide more accurate descriptions of their current situations.

Furthermore, the study was based on comprehensive data about the life-situation 10 years after a first-ever stroke previously not described. The 10-year perspective enabled identification of factors that can be used to predict long-term outcomes at an early stage. After 16 months most of the participants had returned to their own home and the initial functional recovery plateaued, making this a relevant time for assessment of the predictors. Still, it may be objected that the extensive time period between the two follow-ups challenges interpretation of the results. During a 10-year period the consequences of stroke are hard to distinguish from those of ageing or from other disruptive life events (e.g., loss of a spouse, relocation, etc.). Also, developments in healthcare and rehabilitation and changes in society may affect the long-term outcome. Therefore, the predictors should not be interpreted in terms of direct causality but they pinpoint areas that deserve further investigation. Generalizability of the findings to people having a stroke today may be limited due to changes in stroke care or other factors related to this specific sample and assessment period. Hence, more long-term studies in different regions and countries are needed to confirm our results.

Since the purpose of this study specifically concerned social and leisure activities we chose to only use a sub-domain of the FAI, identified in a previous study using ICF-linking [32]. Recognizing that this is not an established approach, we argue that it improved the validity compared to using the total FAI score which includes many other types of activities. We suggest that researchers actively reflect on the content of routinely used instruments such as the FAI. A limitation is that assessments based on the FAI only provide information about the frequency of performing activities. Future studies should also include aspects such as the perceived meaning and value of activities since this likely contributes to why people prioritize certain activities over others. It should also be noticed that the three items of the FAI-CSC only target some aspects of social and leisure activities, and for a more complete picture of engagement in other areas of community, social and civic life, complementing assessments could be considered.

Turning to the statistical analyses, since engagement in social and leisure activities is a complex and dynamic process it was considered important to cover a wide range of potential predictors that represented all four ICF-components [31]. The selection of independent variables was based on previous quantitative and qualitative research reviewed in the Introduction section and on the experiences of the co-authors representing several different disciplines. The four ICF component-specific regression models served to reduce the number of variables included in the final model while allowing for variables from all four components to be represented based on systematic analyses.

Since regression analyses are sensitive to how data is handled, the results of this study were likely affected by the dichotomization of the independent variables. However, all dichotomizations were preceded by extensive discussions in the multidisciplinary research group and aimed to improve interpretability and create statistically sound regression models. For the MMSE and the GDS-20 established cut-off levels were used to create the dichotomized variables. Using the continuous data could have yielded different results, but the results would also have been more difficult to translate to a clinical setting. The same considerations were made regarding the dichotomization of the age variable. Based on the distribution of data, a cut-off at 75 years most clearly demonstrated the age-related differences in activity level. Furthermore, dichotomizing all independent variables facilitates interpretation of the relative weight of each predictor.

### Conclusions

The results indicate that out-of-home mobility and social network resources are likely important factors for the long-term frequency of social and leisure activities after a stroke. Consequently, these aspects deserve further attention in research and interventions aimed at improving the health and well-being of stroke survivors in a long-term perspective. Also, stroke survivors over the age of 75 years should receive special attention due to the higher prevalence of inactivity in this group. Research that further explores the long-term processes related to social and leisure activity after stroke is needed to further explain the findings of this study.
